# Biofilm Formation on Denture Base Material Reinforced With a Novel Organic Material

**DOI:** 10.7759/cureus.65232

**Published:** 2024-07-23

**Authors:** Manoharan P S, Jacob John, Prashanth K, Karavali Prasad, T Mohammed Fahad Ismail, Sneha Sivakumar, Kiruthika Sivakumar, Josephine Flora, Prem Kumar Sivabalan, Priyasha Wase

**Affiliations:** 1 Department of Prosthodontics, Crown and Bridge, Indira Gandhi Institute of Dental Sciences, Sri Balaji Vidyapeeth, Pondicherry, IND; 2 Department of Restorative Dentistry, Dentistry University of Malaya, Kuala Lumpur, MYS; 3 Department of Biotechnology, School of Life Sciences, Pondicherry University, Pondicherry, IND; 4 Department of Biotechnology, Pondicherry University, Pondicherry, IND; 5 Department of Prosthodontics, Adhiparasakthi Dental College and Hospital, Melmaruvathur, IND; 6 Department of Prosthodontics, Crown and Bridge, Mahe Institute of Dental Sciences and Hospital, Mahe, IND

**Keywords:** pmma, biofilm, candida albicans, microcrystalline cellulose, opefb fibers

## Abstract

Background: Microcrystalline cellulose (MCC) is a novel organic material developed by one of the authors in this study. When MCC was incorporated with conventionally available denture base resin, it demonstrated increased flexural strength and flexural modulus. However, it was speculated that because the material is organic, it can promote the growth of *Candida*. The purpose of this study is to evaluate the *Candida albicans* biofilm formation on polymethyl methacrylate (PMMA) denture base resin incorporated with MCC.

Materials and methods: MCC is an organic material extracted from the oil palm empty fruit bunch (OPEFB). The growth of *C. albicans* and biofilm formation in three test groups were compared by biofilm assay and imaging techniques like microscopy (by safranin staining) and scanning electron microscopy. The three test groups were comprised of MCC-reinforced PMMA containing OPEFB fibers of 50-micrometer thickness at 5% weight reinforcement, conventionally and commercially available heat cure PMMA, and an empty well to assess any discrepancies from the environment.

Results: The test groups showed increased biofilm formation by *C. albicans* compared to commercially and conventionally available heat cure PMMA. Reinforcement with MCC showed higher biofilm formation of 1.43 times higher compared to conventional PMMA. Biofilms formed by *Candida albicans* on MCC-reinforced PMMA appeared heterogeneous in structure, comprised of yeast cells and hyphae, surrounded by a higher density of polysaccharide extracellular matrix material compared to that of conventionally available heat cure PMMA.

Conclusion: Biofilm formation is increased in denture base resin incorporated with MCC. More investigation is warranted to study the antifungal efficacy of the addition of antifungal agents to the reinforced denture base resin.

## Introduction

Since 1937, polymethyl methacrylate (PMMA) has been the material of choice for denture bases. The era of denture base resin has attempted through the years to enhance the physical, mechanical, optical, and antifungal properties of this material with rubber, polymeric biocides, fibers (Kevlar, aramid, carbon, nylon, glass, fluoridated glass, polyethylene), fillers (metal, ceramic, silica, hydroxyapatite, mica), nanofiller nanoparticles (silver, silver zeolites, zinc oxide alumina, zirconia, titania, gold, hydroxyapatite, silica, nano clay), nanotubes (carbon, zirconia, titanium dioxide, halloysite), and natural antifungal agents (micro and nanocrystalline cellulose, chitosan, neem, and henna) [1,2].

A novel organic material, microcrystalline cellulose (MCC) derived from oil palm empty fruit bunch (OPEFB), widely prevalent in Southeast Asia, was developed in the recent past by one of the contributors of this study. The extraction of MCC was done by hydrolysis of OPEFB. It was found that 50-micrometer thickness at 5% weight reinforcement significantly enhances the overall flexural strength and flexural modulus of PMMA [3]. It was also proven through cytotoxicity studies that OPEFB-derived MCC did not promote cell death and other toxic effects [4]. Encouraging the circular economy approach, these nature-derived products can be effectively used as reinforcement materials. Apart from dentures, the fibers derived from OPEFB have been recommended to be used in various other applications in the polymer and composite industry [5].

Following the study trail, OPEFB-derived MCC has also raised a concern about their possible effect on biofilm formation on the reinforced denture bases. *Candida* adhesion to tooth and denture or restoration surfaces in the mouth is one of the most essential steps to initiate biofilm formation [6]. *Candida* growth in biofilms on dentures has been strongly associated with denture-related stomatitis. Denture stomatitis can be described as a pinpoint, diffuse, or granulomatous inflammation in patients who wear dentures for a long time period or ill-fitting dentures. Further growth and organization of the biofilm are found to be dependent on the other microbiota and host response to these organisms [7]. Although described as a normal inhabitant of the oral cavity, *Candida* can become proliferative and pathologic under certain circumstances when there is an unfavorable change in the local ecosystem such as a prolonged closed atmosphere and lack of saliva and also decreased immune response in immunocompromised situations [8].

As discussed above, heat-cured conventional PMMA resin is the material that has been most commonly used for the manufacture of various prostheses most commonly employed in dentistry. Although computer-aided designing/computer-aided manufacturing (CAD/CAM) acrylic blocks have replaced the curing technology, the formation of *Candida* biofilms on such surfaces is inevitable. In a study comparing the biofilm formation occurring on a conventional PMMA to CAD/CAM polymer denture materials, it was seen that biofilm formation was significantly higher in the conventional PMMA [9].

From the viewpoint of microbial biofilm retention and stain deposition, the prosthesis is expected to present a smooth surface. However, in dentures, the intaglio surface of the denture should not be polished as it would abrade the surface details making the surface smooth and glossy thereby leading to loss of retention. Hence an unpolished surface is a potential surface for biofilm formation. Various surface modifications like shell blasting and viscoelastic shot blasting are done to minimize surface roughness and also to maximize wetting at the same time. In an in vivo/in vitro study, it was found that surface roughness and biofilm formation were minimized with viscoelastic shot blasting [10]. However, for practical purposes, all dentures are left unpolished and untouched with minimal adjustments like removal of nodules and macro irregularities to prevent loss of retention.

With the background of the above, it is clear that biofilm formation on denture bases fabricated with common material like PMMA is of genuine concern even today. With the development of new materials, apart from mechanical and physical properties, biofilm formation should also be studied. This is a commonly neglected entity with scant evidence in literature. This developed novel material was studied for cytotoxicity and strength-related properties. The study on biofilm formation on the denture base reinforced with this material would throw light on the acceptability of material or development of material further to overcome any drawbacks identified.

## Materials and methods

A stainless-steel disc with a through central relief of standard size of 10 x 1 mm was fabricated [11]. Wax patterns for the specimens to be prepared were made in the stainless-steel die. They were then invested in a flask and invested with gypsum, they were placed in a water bath at room temperature and the curing temperature was programmed to 74°C for one and a half hours, followed by 100°C for one hour. As a result of the heat, the wax melted but the outline of the discs was present. This mold was then packed with heat cure PMMA resin for half of the samples and the other half with MCC incorporated into PMMA at 5% weight. They were subsequently subjected to the conventional short heat curing cycle. Processing was performed in a water bath at 74°C for two hours and increasing the temperature to 100°C for one hour. Following this, the flask was opened and the discs obtained were trimmed and polished on one side, while the other side was left unpolished to simulate oral conditions. After preparation, all discs were cleaned and stored in distilled water. The PMMA and MCC-PMMA samples were then divided into groups to carry out a biofilm formation assay, followed by imaging analysis using compound microscopy (by safranin staining) and scanning electron microscopy (SEM).

Biofilm production evaluation using microtiter plate assay

**Figure 1:**
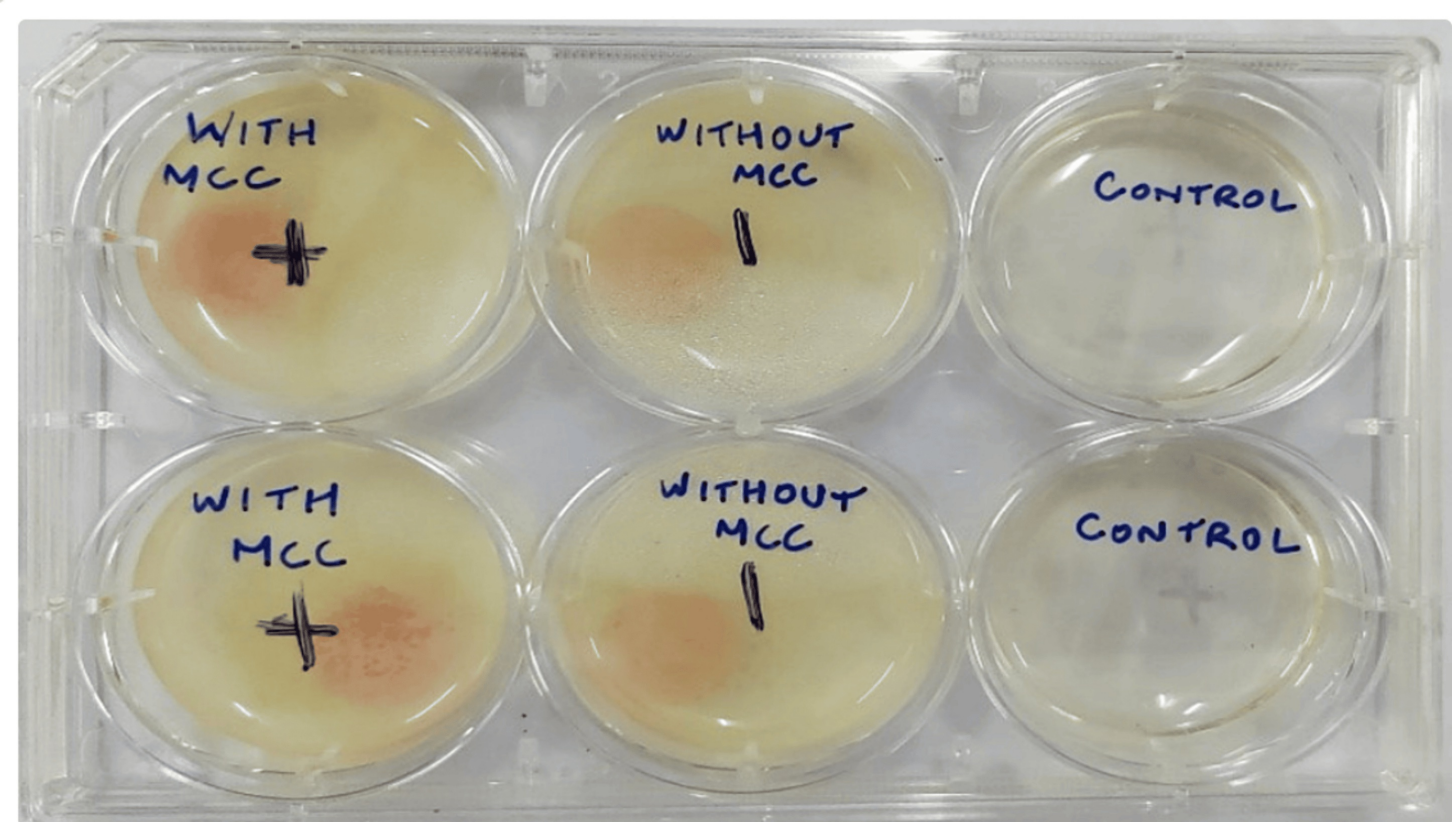
Incubation of C. albicans on discs for 24 hours and 48 hours. A control well was added to check for any contamination of the wells.

Clinical isolate of *Candida albicans* 125 was obtained from the culture collection of the Department of Microbiology, Pondicherry Institute of Medical Sciences, Puducherry, and it was used in the present investigation. *C. albicans* was first inoculated on potato dextrose broth (PDB) (SRL Chemicals, Mumbai, India) and incubated at 37°C for 24 hours. All the discs were sterilized by autoclaving at 121°C for 15 minutes at 15 lbs of pressure. MCC-incorporated PMMA resin discs were placed in a 6-well plate. Conventional PMMA resin discs were also placed in a 6-well plate and maintained as a control. Additionally, empty wells without any addition were used as a negative control. All the groups and positive and negative controls were performed in triplicates. Further, 5 ml of PDB was added to all the wells and 20 µl of *C. albicans* overnight culture was taken and inoculated into these wells and then incubated at 24 hours and 48 hours (Figure 1).

Microtiter plate assay was used to determine the quantitative estimation of biofilms formed according to the method described elsewhere [12]. After the incubation period of 48 hours, the biofilm formation on the discs was evaluated. All the discs were taken out from the 6-well plates with the help of sterile forceps and transferred to a new 6-well plate. Biofilm on the discs was washed with 33% of glacial acetic acid. The washed biofilm was treated with 200 µl of 0.1% crystal violet. Then the 200 µl of the washed biofilm crystal violet complex was transferred to a fresh 96-well plate and the optical density was measured at 595 nm with a microplate reader (Bio-Rad Microplate Reader 550, Hercules, CA). Readings from the triplicate samples were taken and the mean and standard deviation were determined.

Staining of biofilm on PMMA discs

Safranin was used as a stain for visualization of the biofilms of *C. albicans* on the polymethyl acrylate incorporated with and without MCC under a compound microscope. The *C. albicans* biofilm grown on polymethacrylate with and without MCC was scrapped using sterile forceps and it was spread on a clean glass slide and then heat fixed. The slides were stained with safranin (Nice Chemicals (P) Ltd, Kerala, India) for one minute and then excess stain was removed by washing with distilled water and the slide was air dried. The air-dried slides were observed in a compound microscope, under 100x magnification with oil immersion.

Scanning electron microscopy

The *C. albicans* biofilms formed on the discs were washed with phosphate buffer solution (PBS) and then the discs were immersed in PBS-containing tubes. Subsequently, discs containing tubes were subjected to ultrasonication in pulses (30 seconds on and off) for 10 minutes. After the sonification, the discs were removed and cell suspension of PBS was centrifuged at 1000 rpm for 10 minutes. Then supernatant was discarded and the pellet was collected for further investigation. The pellet was then put on the grid and the sample was prepared for the SEM examination that was performed at different magnifications (SEM, Hitachi S-3400N, Hitachi Science Systems, Chiyoda, Japan).

## Results

**Figure 2:**
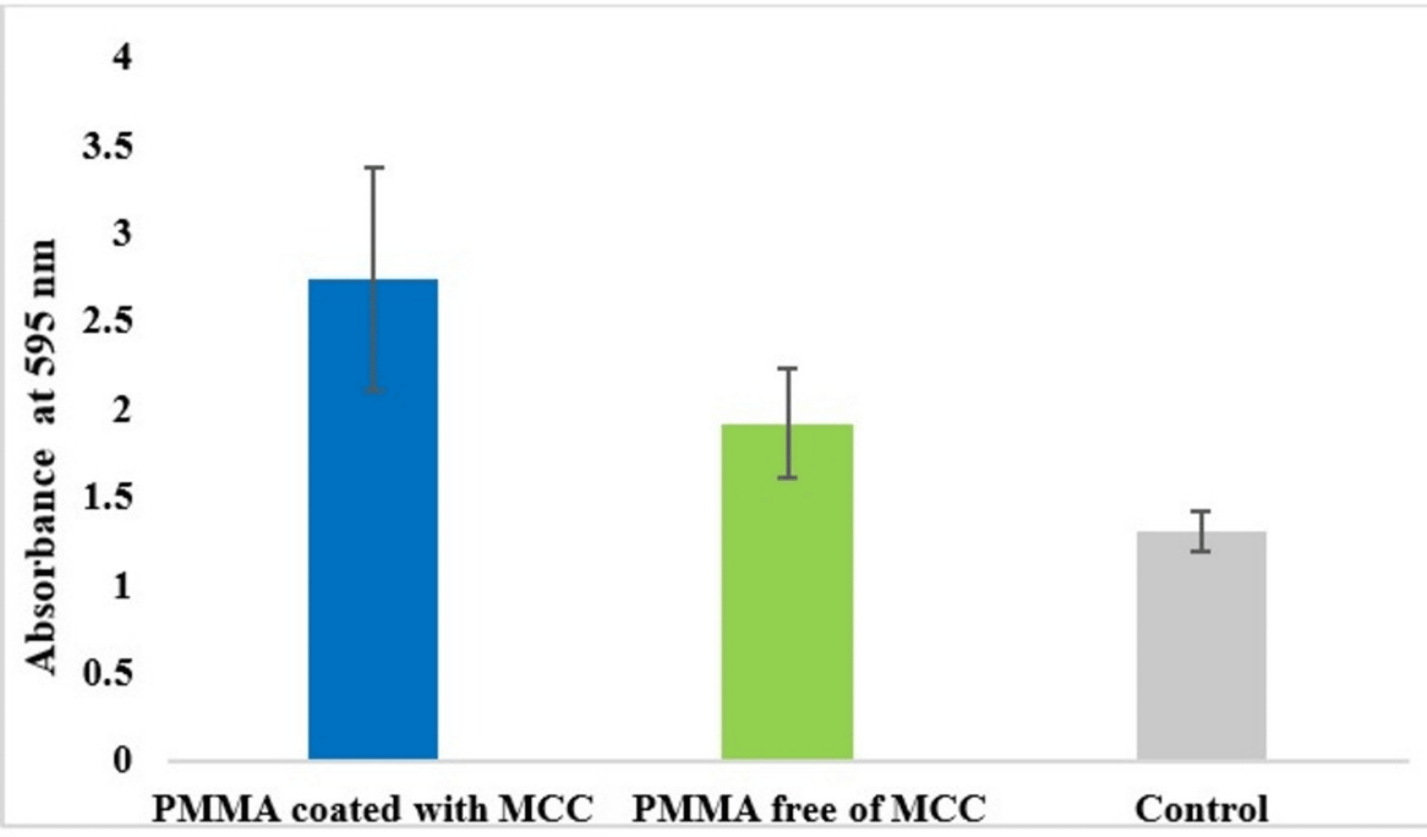
Formation of biofilms by C. albicans. PMMA: polymethyl methacrylate; MCC: microcrystalline cellulose.

**Figure 3:**
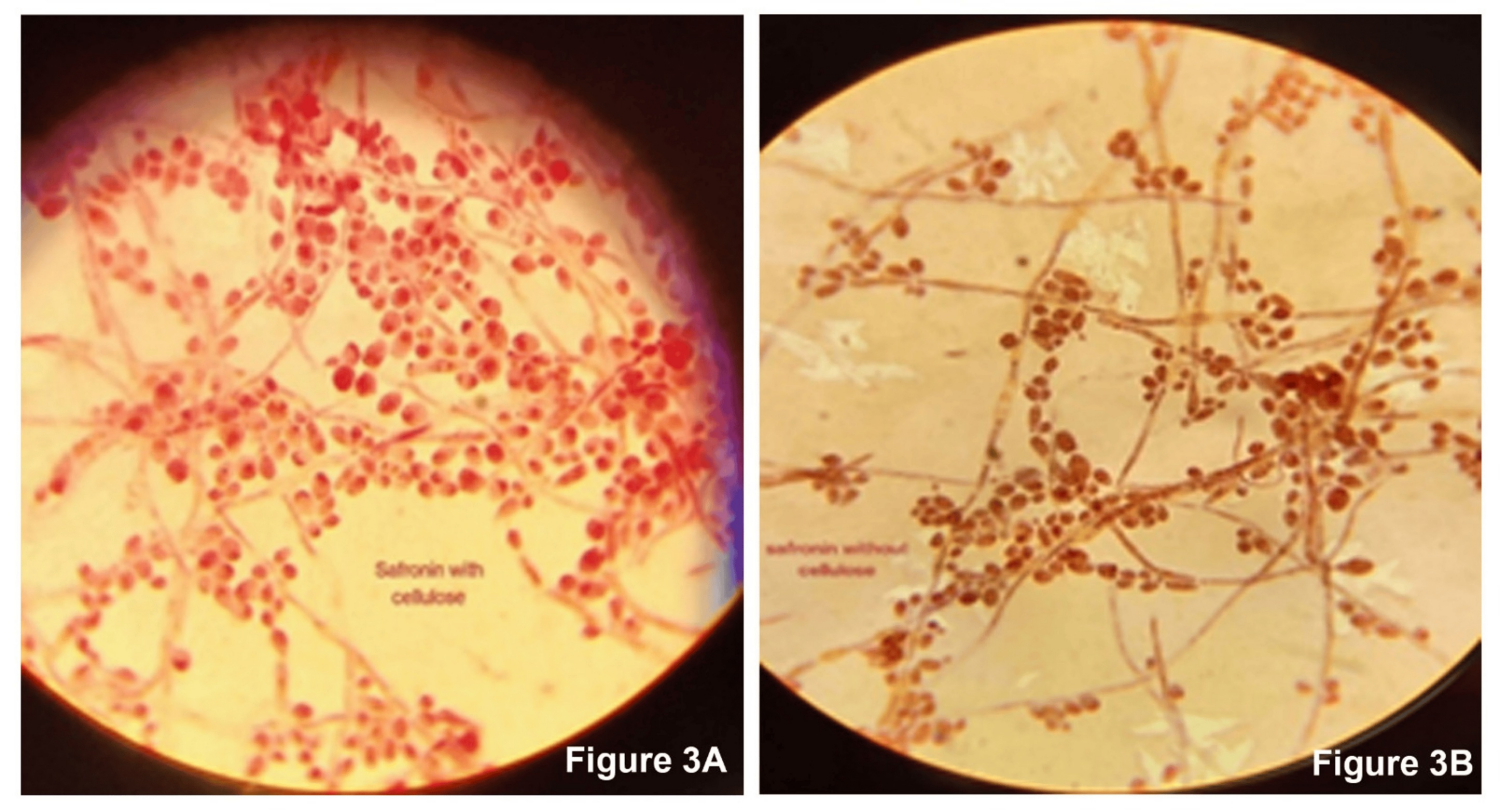
Visualization of biofilms of Candida albicans of (A) polymethyl methacrylate with microcrystalline cellulose and (B) polymethyl methacrylate without microcrystalline cellulose.

Overnight cultures of *C. albicans* that were grown on the discs placed in the wells after the incubation of 24 hours and 48 hours are shown in Figure 1. The quantitative estimation of biofilm formation done by the microtiter plate assay revealed that the biofilm formation by *C. albicans* on the polymethyl acrylate incorporated with MCC was about 1.43 times higher when compared to the polymethacrylate free of MCC. The absorbance of biofilms at 595 nm that was performed in triplicates was averaged and tabulated in Table 1 and the same is illustrated in Figure 2. Safranin staining of biofilms formed on the disc showed the *C. albicans* cells in pink color along with the pseudo hyphae. Extracellular polysaccharide (EPS) matrix accumulated around the cell wall that was stained with safranin was clearly observed. The biofilms (EPS) around the cells of *C. albicans* grown on the PMMA with MCC showed a density that was much higher when compared to the biofilms grown on the polymethacrylate free of MCC (Figure 3).

**Table 1:** Formation of biofilms by C. albicans. PMMA: polymethyl methacrylate; MCC: microcrystalline cellulose.

Biofilms	OD at 595 nm absorbance units (AU), mean ± SD values
PMMA coated with MCC	2.74 ± 0.63
PMMA free of MCC	1.91 ± 0.31
Control	1.30 ± 0.11

Scanning electron microscope

**Figure 4:**
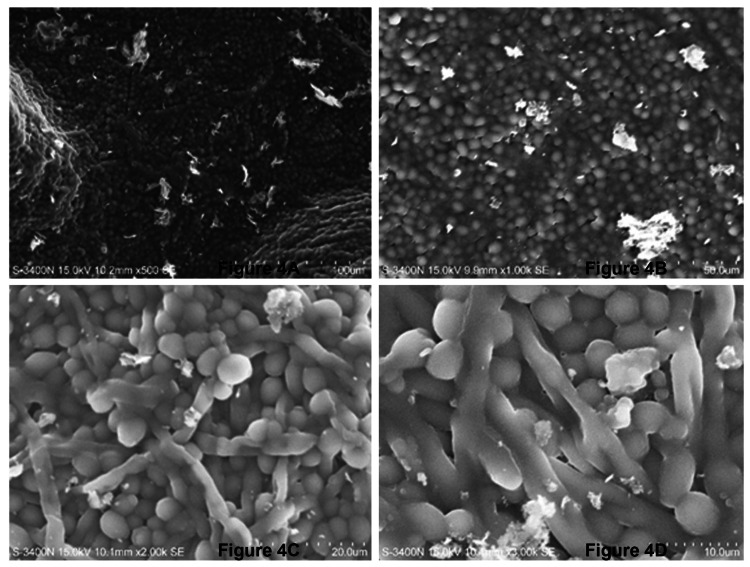
SEM images of C. albicans biofilms formed on MCC-PMMA discs. (A) 500x, (B) 1000x, (C) 2000x, and (D) 3000x of magnification. SEM: scanning electron microscopy; PMMA: polymethyl methacrylate; MCC: microcrystalline cellulose.

**Figure 5:**
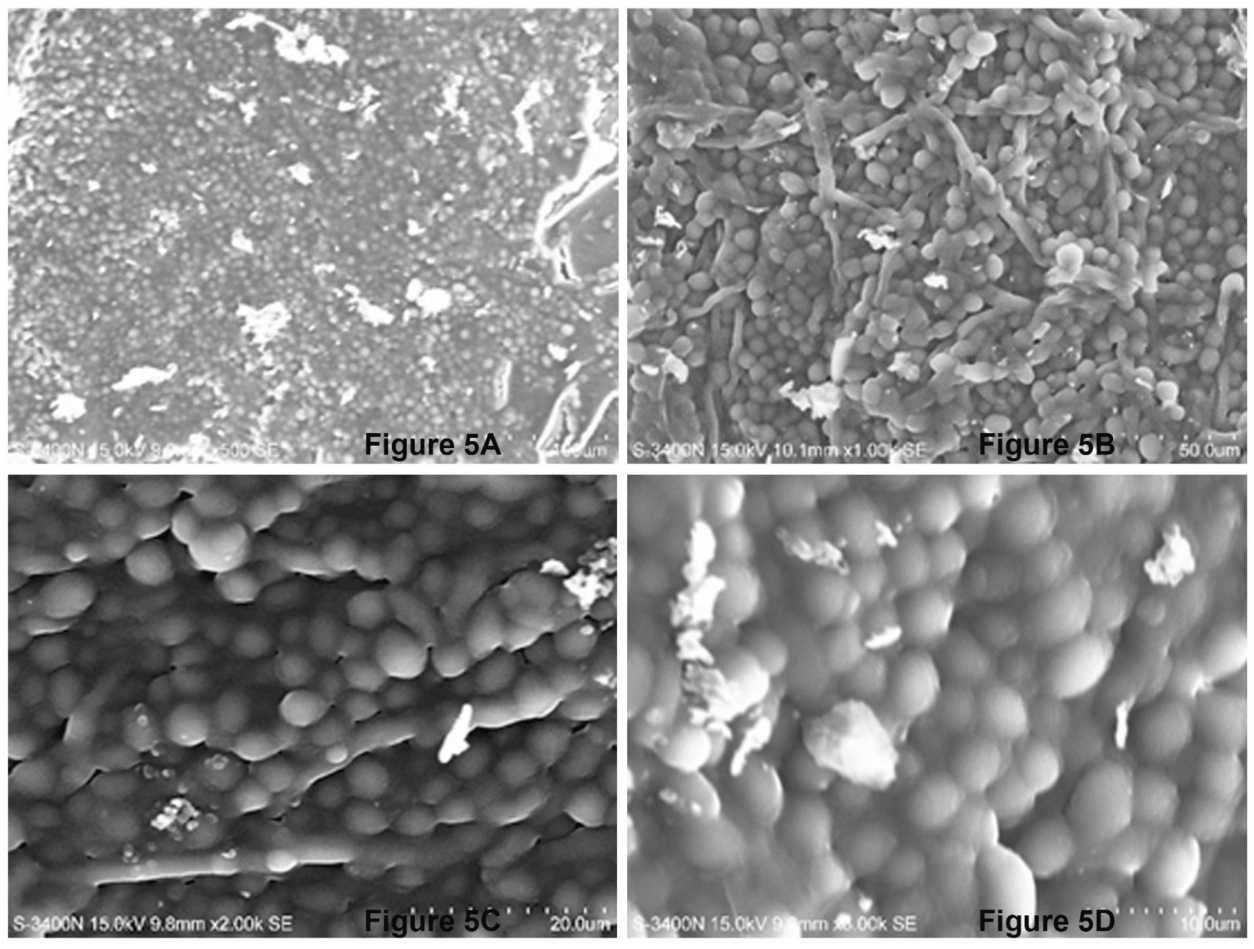
SEM images of C. albicans biofilms formed on PMMA discs free of microcrystalline cellulose. (A) 500x, (B) 1000x, (C) 2000x, and (D) 3000x of magnification. SEM: scanning electron microscopy; PMMA: polymethyl methacrylate.

SEM images revealed biofilm formation was more mature in MCC-PMMA in comparison with the conventional PMMA (Figures 4, 5). Biofilms formed by *C. albicans* on MCC- PMMA appear to be more heterogeneous in its structure and it was comprised of yeast cells and hyphae, which were surrounded by polysaccharide extracellular matrix material. These structures were forming scaffolds through adhesion of cells and hyphae at different levels of the surfaces showing a vertical growth (Figure 4A), which was not observed in the SEM images of PMMA free of MCC resin (Figure 5A). In higher magnification, one can witness that *C. albicans* biofilms are composed of yeast form of cells entangled with hyphae forming a stacking multilayer structure intimately packed with clusters of cells and hyphae (Figure 4D). Matured biofilm (Figure 4) is shown to bear a thick layer of EPS wherein, yeast cells and hyphal forms form a dense network. Such kind of biofilm development was not observed in the conventional PMMA (Figure 5). Moreover, hyphal structures were totally absent in MCC free of cellulose.

## Discussion

In this study, the MCC used was derived from OPFEB through hydrolysis and ground to fine powder with uniform particle size. The cellulose was already optimized and proven to be effective at 50 micrometers and 5% weight by one of the authors in the study. Flexural strength, flexural modulus, and cytotoxicity were studied and it was found to produce better results than conventional PMMA resin [3,4]. Other incorporations like silver nanoparticles to impart antimicrobial effect have reduced the strength of the conventional PMMA [13]. Surface roughness plays an important role in the adherence of biofilms. Bacterial and fungal adhesion on the acquired pellicle is the first stage in the formation of oral biofilms [14]. As the biofilm matures, hyphal formation is an important indicator that can cause tissue damage and stubborn colonization and maintenance of biofilms [15].

All PMMA specimens were prepared uniformly with standardized protocol in this study. The intaglio surface is left unpolished to simulate the acrylic intaglio surface of the denture. The samples were then randomized across groups to avoid the allocation of specimens consciously and blinded to the investigator. Many methods were used in the literature to study biofilm formation. We have used biofilm assay to calculate the quantitative amount of biofilms formed, safranin staining was used to characterize the quantity and quality of *C. albicans* growth and SEM to describe the fungal growth over a period of time in various magnifications. All the specimens were subjected to all the three kinds of tests performed. Care is taken to prevent contamination of specimens. A control well was dedicated to evaluate that the tests were conducted in a sterile environment. All through the study, the negative control group showed no growth of fungus.

The growth of *C. albicans* species was prominent with longer mycelia and dense colonies. The condition optimization allowed the investigator to limit the number of specimens used. In our study, we chose SEM over others, as our long-term objective is to develop a stronger PMMA that may have less biofilm absorption on the surface as compared to conventional PMMA. SEM has been considered one of the best methods for visualization and description of biofilm morphology. This method is also found to be highly co-relatable compared to other methods. This method also allows a wider range of magnifications (20 to 30000x). Our study used 500x, 1000x, 2000x, and 3000x magnifications, which are adequate to visualize biofilm morphology at different levels. However, confocal laser scanning microscopy (CLSM) visualizes biofilm in its three-dimensional architecture and its time-dependent variation (four-dimensional real time), which is a better method [16].

Overall, the results of the study revealed that the MCC-PMMA group demonstrated an increase in the growth of *C. albicans* species. Biofilm is an unavoidable entity and it was anticipated that incorporating a plant-derived cellulose kind of material can demonstrate increased fungal growth. Some studies conducted last year demonstrated some improvement in terms of biofilm reduction with the incorporation of cellulose nanocrystals [17]. This enables us to further expand the scope of the study by modifying the material or incorporating antimicrobials/antifungals in the denture base material. Silver, silver nano, calcium, silane coupling agents, chitosan, neem, etc. have been tried in the past to provide antimicrobial effects inside the oral cavity. Many particles that were incorporated were found to be reducing the mechanical properties. More studies are needed to bring out the effectiveness and safety, including assessment of biofilm formation. This study can be claimed as unique, as it is one of the few studies investigating biofilm formation on acrylic surfaces by incorporating fibers, fillers, etc.

The limitations of the study are that only one strain of *C. albicans* was used and we used it in only one type of denture base resin - conventional PMMA. Most studies in the literature focus on *Candida* biofilm formation on dentures and restorative materials. Conventional PMMA is commonly used as a denture base material all over the world. However, there are studies of biofilm formation over flexible denture base materials and reline acrylic materials, which is beyond the scope of our aim and objectives.

## Conclusions

Despite some limitations, this study was able to conclude that PMMA reinforced with MCC showed increased biofilm formation by *Candida albicans* when compared to the conventional heat cure PMMA. Besides, matured biofilm growth by *Candida albicans* that was characterized by a heterogeneous structure with hyphal growths at different levels vertically was observed on PMMA reinforced with MCC, which was not observed in heat cure PMMA. The results of the present study indicate that MCC-incorporated PMMA warrants further studies to look into the scope of the addition of antifungal agents or methods to improve biofilm resistance.
